# Uncommon features of surgically resected ALK-positive cavitary lung adenocarcinoma: a case report

**DOI:** 10.1186/s40792-017-0322-2

**Published:** 2017-03-20

**Authors:** Shinkichi Takamori, Masafumi Yamaguchi, Kenichi Taguchi, Makoto Edagawa, Shinichiro Shimamatsu, Ryo Toyozawa, Kaname Nosaki, Fumihiko Hirai, Takashi Seto, Mitsuhiro Takenoyama, Yukito Ichinose

**Affiliations:** Department of Thoracic Oncology, National Kyushu Cancer Center, 3-1-1 Notame, Minami-ku, Fukuoka, 811-1395 Japan

**Keywords:** Anaplastic lymphoma kinase, Cavitary mass, Radiological feature

## Abstract

Some features found on chest computed tomography (CT), such as central tumor location, large pleural effusion, and the absence of a pleural tail, and a patient age of less than 60 years, have been suggested to be useful in predicting *anaplastic lymphoma kinase* (*ALK*) rearrangement in patients with non-small cell lung cancer (NSCLC).

A 68-year-old female patient with a history of gynecological treatment was found to have a cavitary mass in the right lower lobe on an annual chest roentgenogram. The tumor was located in the peripheral area with a pleural tail showing no pleural effusion. In addition, two pure ground-glass-opacity nodules (p-GGNs) in the right upper lobe of the lung were detected on consecutive chest CT scans. The patient underwent right lower lobectomy, partial resection of the right upper lobe, and hilar mediastinal lymph node dissection for complete resection of each tumor. The pathological diagnosis was invasive mucinous adenocarcinoma with signet-ring cells for the cavitary mass in the right lower lobe and invasive adenocarcinoma for the rest of the p-GGNs; subcarinal lymph node metastasis was also detected. The *ALK* rearrangement was detected by fluorescence in situ hybridization from the cavitary mass. The patient underwent four cycles of cisplatin and vinorelbine chemotherapy as standard adjuvant chemotherapy for pStage III NSCLC. The *ALK* fusion gene status of NSCLC with atypical CT features should also be investigated.

## Background

Several radiological features delineated by chest computed tomography (CT) of non-small cell lung cancer (NSCLC) have been reported to be associated with some utility in detecting the gene mutation status, such as *epidermal growth factor receptor* (*EGFR*) or *anaplastic lymphoma kinase* (*ALK*) rearrangement. Certain features of a tumor on chest CT, such as convergence, notch, and a pleural tail, have been reported to have a significant association with an *EGFR* or *ALK* fusion gene mutation [1–5]. With regard to the mutation of the *ALK* fusion gene, the combination of certain tumor features on chest CT, such as a central tumor location, large pleural effusion, and the absence of a pleural tail, and a younger patient age (<60 years old), were shown to have a significant relationship with *ALK* rearrangement in patients with NSCLC [6]. In general, larger squamous cell carcinomas often show cavitation, and cavitary masses have been found in a number of adenocarcinoma patients who had a poor prognosis [7].

We herein report a case of surgically resected lung adenocarcinoma that presented as a cavitary mass harboring *ALK* fusion with rare radiological features.

## Case presentation

A 68-year-old female was found to have a cavitary mass on annual chest roentgenography in December 2015. She had a history of treatment for cervical cancer in the uterus in 2001 and had been followed up at our hospital without recurrence. She was a never-smoker and had no significant abnormalities on a physical examination except for an abdominal scar related to the treatment for her cervical cancer.

**Fig 1:**
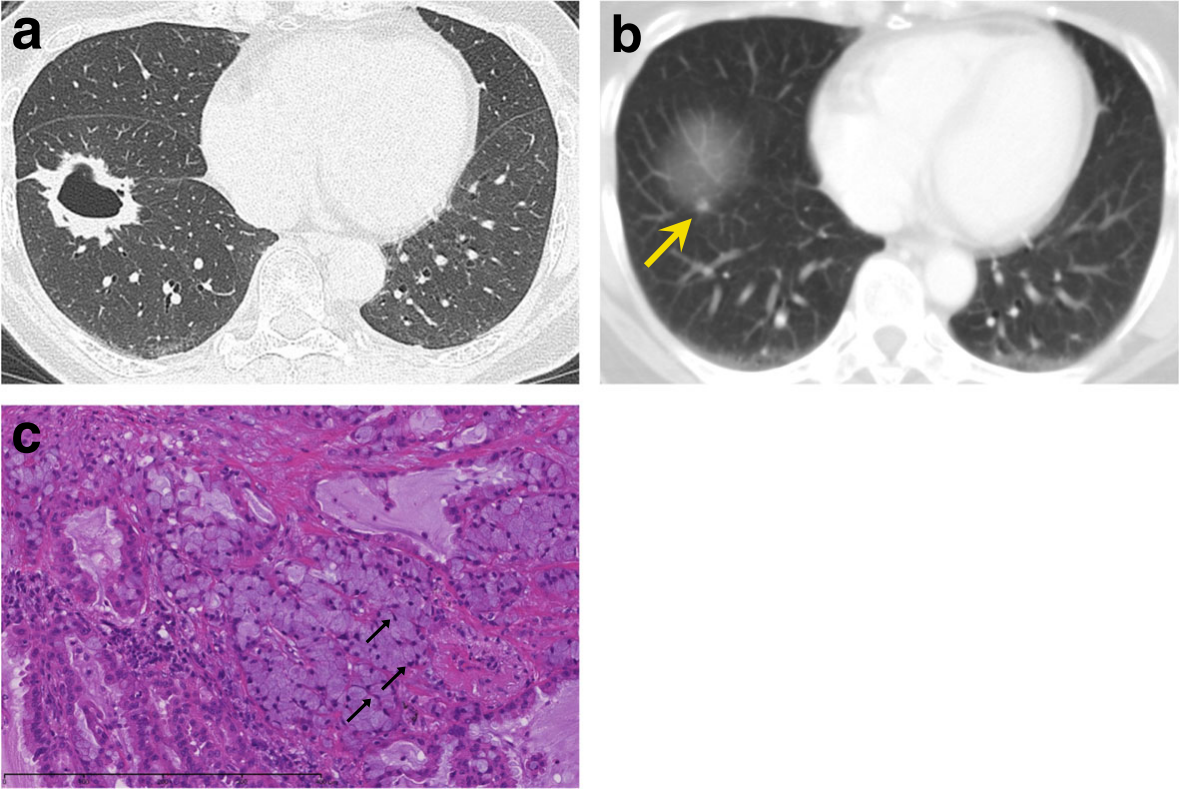
**a** Chest cross-sectional computed tomography (CT) showing a cavitary mass in the S6 segment in the peripheral area with a pleural tail and without pleural effusion. **b** An abdominal CT scan taken in 2008 occasionally showed a tiny nodule on the basal segment of the *right* lower lobe adjacent to the visceral pleura on the diaphragm (*arrow*). **c** The results of a histological examination. Invasive mucinous adenocarcinoma with signet-ring cells in an S6 tumor (hematoxylin and eosin staining, original magnification ×400)

Chest CT revealed a cavitary mass, approximately 4.3 cm in its maximum dimension, in the basal segment of the right lower lobe of the lung (Fig. 1a). In addition, we noted 2 pure ground-glass-opacity nodules (p-GGNs), 8 and 12 mm in their maximum dimensions. Retrospectively, abdominal CT performed in 2008 occasionally revealed a tiny nodule in the basal segment of the right lower lobe adjacent to the visceral pleura on the diaphragm (Fig. 1b). No significant hilar or mediastinal lymph node swelling was observed. fluorodeoxyglucose positron emission tomography (FDG-PET) showed no abnormal accumulation except in the mass in the right lower lobe. A biopsy of the tumor via fiber optic bronchoscopy could not distinguish the cavitary mass from a metastatic or primary tumor. The two p-GGNs were considered to be multiple primary lung cancers. The patient underwent video-assisted thoracoscopic right lower lobectomy, partial resections for the two p-GGNs in the right upper lobe, and systemic hilar mediastinal lymph node dissection as curative intent resection. An analysis of the intraoperative frozen sections for the three tumors revealed adenocarcinoma.

A pathological examination revealed the cavitary mass in the right lower lobe to be invasive mucinous adenocarcinoma (60% acinar, 30% micropapillary, 10% papillary growth pattern) with signet-ring cells (Fig. 1c). Mediastinal lymph node metastasis was found in the subcarinal lesion. The two p-GGNs were pathologically different from the cavitary mass in the right lower lobe; thus, the pathological diagnosis was multiple primary NSCLC: pT2aN2M0 stage IIIA adenocarcinoma for the cavitary mass in the right lower lobe and pT1aN0M0 stage IA adenocarcinoma for each p-GGN in the right upper lobe. The patient received four cycles of cisplatin and vinorelbine as adjuvant chemotherapy. In addition, *ALK* rearrangement was detected by FISH from the specimen obtained from the cavitary mass on the right lower lobe. The postoperative course was uneventful, and the patient is currently disease-free at 5 months post operation.

### Discussion

Previous reports have shown that some CT features are significantly associated with the *ALK* fusion gene [2–5]. Yamamoto et al. reported that three CT features, namely a central tumor location, large pleural effusion, and the absence of pleural tail, with a patient age of less than 60 years, were good predictors of the presence of the *ALK* fusion gene in cases of NSCLC [6]. In addition, Yamamoto et al. attempted to predict the presence of the *ALK* fusion gene using those three CT features and the age of patients and revealed a sensitivity and specificity of 89.4 and 100.0%, respectively, for the presence of the *ALK* fusion gene in certain cohorts of patients. However, the equation failed to demonstrate the presence of the *ALK* fusion gene in our patient in the present report. Retrospectively, a tiny nodule was occasionally found in the basal segment adjacent to the visceral pleura (the peripheral area of the right lung on an abdominal CT taken 8 years prior as a follow-up of cervical cancer of the uterus; Fig. 1b). The present case therefore differed with respect to the predictive factors for the presence of the *ALK* gene fusion in the cavity formation in the tumor, an initial tumor location in a peripheral site without pleural tail formation, and a patient age over 60 years old.

With regard to the pathological features of this case, the permanent specimen showed invasive mucinous adenocarcinoma with signet-ring cells (Fig. 1c). Signet-ring cells are a rare feature of primary lung adenocarcinoma [7]; the presence of signet-ring-cell elements is significantly more frequent in *ALK*+ lung cancer [8]. Given that the *ALK* status of the patient was positive, these findings were consistent with those of previous reports.

## Conclusions

We report a case of NSCLC harboring *ALK* rearrangement with uncommon radiological features. The *ALK* gene status of NSCLC with atypical CT features should also be investigated.

## Abbreviations

*ALK*: Anaplastic lymphoma kinase
CT: Computed tomography
EGFR: Epidermal growth factor receptor
FDG-PET: Fluorodeoxyglucose positron emission tomography
NSCLC: Non-small cell lung cancer
p-GGNs: Pure ground-glass-opacity nodules
